# Abdominal Hypertrophy Syndrome: Characteristics and Potential Pathophysiology

**DOI:** 10.7759/cureus.72026

**Published:** 2024-10-21

**Authors:** Igor A Suslin, Iakov V Efimenko, Ricardo Castrellon, Tarik M Husain

**Affiliations:** 1 Plastic Surgery, Larkin Community Hospital, Miami, USA

**Keywords:** abdominal hypertrophy syndrome, abdominal organomegaly, anabolic steroids, bodybuilder gut, bubble gut, growth hormone, hgh gut, palumboism, steroid gut, visceral adiposity

## Abstract

Abdominal hypertrophy syndrome, known as steroid gut, is an uncommon condition affecting bodybuilders and athletes engaged in prolonged usage of growth hormone (GH), insulin, and other anabolic agents. The condition is more commonly known in the professional bodybuilding community as Palumboism, named after David Palumbo, an American bodybuilder. It is characterized by significant enlargement and distension of the abdomen. Precise pathophysiological mechanisms and underlying causes of Palumboism have yet to be fully understood. The primary objective of this study is to conduct a comprehensive literature review of the condition and explore the pathophysiology and possible treatment modalities. We aim to contribute to the existing knowledge of Palumboism and lay the foundation for clinical and surgical management. A literature review was conducted using PubMed and other sources. Specific keywords, such as "palumboism," "bodybuilder gut," "steroid gut," "HGH gut," "insulin gut," "bubble gut," "muscle gut," "abdominal distension," "abdominal organomegaly," "visceral adiposity," "abdominal obesity," "anabolic steroids," and "growth hormone," were employed to retrieve relevant articles. The inclusion criteria focused on studies that investigated the pathophysiology, clinical presentation, and management of Palumboism. A total of 1,222 studies were identified through the search criteria, of which 451 were screened, 33 were assessed for eligibility, and 30 studies were included in the final review. Literature review revealed that no peer-reviewed studies dedicated to Palumboism, underscoring the insufficient research conducted in this area. The available anecdotal data suggest that the prolonged use of high-dose anabolic steroids, particularly human GH and insulin, may contribute to the development of Palumboism. Several potential mechanisms have been proposed, including visceral adiposity, organomegaly, and altered collagen synthesis. Given the dearth of available research on Palumboism, a comprehensive understanding of this condition is yet to be established. Further studies are warranted to elucidate the pathophysiology, establish diagnostic criteria, and explore treatment options for affected individuals.

## Introduction and background

Palumboism, colloquially referred to as steroid gut, represents a distinctive condition characterized by the development of a prominently enlarged and distended abdomen among bodybuilders and athletes who engage in prolonged anabolic steroid usage. Despite its widespread recognition within the bodybuilding and fitness community, Palumboism does not hold official recognition as a medical term or a specific diagnosis in the realm of medical literature. Notably absent are formal classification and diagnostic criteria, resulting in its omission from standard medical textbooks and the authoritative classifications presented by organizations such as the World Health Organization (WHO) and the American Medical Association (AMA).

While Palumboism serves as a well-known concept in certain circles, medical professionals conventionally employ the terms "abdominal enlargement" or "abdominal distension" to depict the visible outcome observed in individuals immersed in extended anabolic steroid utilization. In scientific and medical literature, more encompassing descriptions may emerge, such as "abdominal organomegaly" or "visceral adiposity," focusing on the physiological shifts underlying prolonged steroid exposure rather than relying on the specific label of Palumboism.

The emergence of what is colloquially termed "bubble gut," or Palumboism, among bodybuilders since its introduction in the 1990s has emerged as a notable concern within the fitness community. This issue significantly influences the aesthetic qualities of athletes and bodybuilders alike. Despite its prevalence, a consensus pertaining to medical or surgical management of this condition remains elusive.

While the precise etiology of this phenomenon remains enigmatic, it is hypothesized that various contributing factors synergistically culminate in abdominal distension and enlargement. A conspicuous element influencing bubble gut is the utilization of human growth hormone (HGH) among bodybuilders, frequently in doses that far exceed typical therapeutic ranges. Elevated HGH levels have the potential to induce excessive tissue expansion, including within the intestines, thereby leading to a substantial increase in abdominal size. Studies examining the effects of overexpressing bovine growth hormone (GH) in transgenic mice have revealed marked increases in the weight and length of the small bowel, accompanied by a 50-100% amplification in mucosal mass [1]. This evidence is reinforced by additional investigations demonstrating elevated villus content of IGF-1 mRNA in the bowel of GH transgenic mice compared to their wild-type counterparts, supporting the notion of localized IGF-1 production stimulated by GH. Moreover, in rats subjected to substantial (75-80%) small intestinal resection, both hGH and IGF-1 were found to enhance the postoperative hyperplasia evident in the ileal remnant [2,3].

In the context of humans, GH, or somatotropin, engages its membrane-bound growth hormone receptor (GHR) situated on hepatocytes, subsequently activating the cytoplasmic tyrosine kinase Janus Kinase 2 (JAK-2). This activation leads to tyrosine phosphorylation within both the JAK-2 enzyme and GHR [4,5]. Consequently, the liver secretes insulin-like growth factor-1 (IGF-1), or somatomedin, which binds to its receptor on colonic epithelial cells, orchestrating heightened epithelial proliferation, reduced apoptosis, and intensified angiogenesis [6]. This relationship between extensive epithelial proliferation and a broad proliferation zone with the levels of GH and IGF-1 is evident in the colons of acromegalic patients [7]. Acromegaly, characterized by excessive GH production and resulting in progressive somatic disfigurement and systemic manifestations [8], potentially shares intestinal pathophysiological mechanisms with Palumboism.

While prior research has extensively explored the utilization of anabolic-androgenic steroids (AAS), including testosterone and its derivatives [9,10], limited attention has been dedicated to other frequently used performance-enhancing drugs (PEDs) like GH, insulin-like growth factor-I (IGF-I), and insulin itself. In 1992, a study disclosed that roughly 5% of male high school students acknowledged experimenting with GH during their high school athletic careers, with a notable portion being acquainted with peers similarly involved in GH usage [11]. Moreover, among middle-aged and elderly individuals, the pursuit of GH administration aims to augment muscle mass and recapture youthful physical attributes. The pervasiveness of PED misuse extends to the weightlifting community, as evidenced by a survey of 231 weightlifters where 12% admitted to historical and/or current GH or IGF-I usage, with a substantial 80% of these respondents exhibiting indications of prior or ongoing AAS dependence [12]. Another noteworthy addition to PEDs is insulin, frequently adopted by bodybuilders due to its purported anabolic effects, including the vital stimulation of glycogen synthesis for post-exercise muscle recovery.

## Review

To comprehensively investigate Palumboism, an extensive and rigorous literature review was conducted. The primary objective was to gather a broad range of relevant studies pertaining to this condition. To achieve this, a systematic search strategy was employed, utilizing the PubMed database and Google Scholar search engine.

To ensure a comprehensive search, a set of carefully selected keywords was employed. These keywords were chosen based on their relevance to Palumboism and its associated features. The specific terms used in the search included "palumboism," "bodybuilder gut," "steroid gut," "HGH gut," "HGH bloat," "insulin gut," "bubble gut," "muscle gut," "abdominal distension," "abdominal organomegaly," "visceral adiposity," "abdominal obesity," "anabolic steroids," and "growth hormone."

By incorporating these diverse keywords, we aimed to capture a wide array of studies and articles that explored various aspects of Palumboism. The intention was to encompass research related to the pathophysiology, clinical presentation, and management of this condition. Through the utilization of these keywords, we sought to identify studies that specifically addressed the mechanisms underlying Palumboism, the characteristic clinical manifestations observed in affected individuals, and the strategies employed for its management.

The search process involved careful examination and evaluation of each retrieved article to determine its relevance and suitability for inclusion in the literature review. The exclusion criteria consisted of studies that were not peer-reviewed, involved non-adult populations, were duplicates, were not in English, and did not comply with the intended clinical presentation. Only studies that directly addressed the pathophysiology, clinical presentation, and management of Palumboism were selected for further analysis and inclusion in the review. A total of 1,222 studies were identified through the search criteria, of which 451 were screened, 33 were assessed for eligibility, and 30 studies were included in the final review.

During the literature review, it became apparent that there is a scarcity of studies dedicated to investigating Palumboism. The existing body of literature primarily comprises anecdotal evidence, indicating a significant research gap in this field. The limited availability of substantial research underscores the need for further investigation into this condition.

Based on the available evidence, it is suggested that the prolonged and high-dose usage of anabolic steroids, particularly GH and insulin, may play a role in the development of Palumboism. However, the current understanding of the underlying mechanisms is extremely limited.

Several potential mechanisms have been proposed to explain the abdominal distension observed in individuals with Palumboism.

Cushing’s syndrome

Mechanisms of visceral obesity in Cushing's syndrome are related to glucocorticoids’ regulative action on adipose-tissue differentiation, function, and distribution. In excess, glucocorticoids cause central obesity. Adipose stromal cells from omental fat, but not subcutaneous fat, can generate active cortisol from inactive cortisone through the expression of enzyme 11 beta-HSD1, the expression of which is increased further after exposure to cortisol and insulin [13]. Glucocorticoids are regulated by adrenocorticotropic hormone (ACTH). In one controlled study examining the increase of reinforcing value of exercise as opposed to drug-induced euphoria in athletes using anabolic-androgenic steroids (AAS), the AAS-using groups had significantly higher ACTH levels than heavy exercising controls [14]. These mechanisms in part can hypothetically contribute to the development of abdominal distension in athletes and bodybuilders.

GH

Organomegaly, which refers to the abnormal enlargement of organs, has been postulated as another contributing factor to Palumboism. The prolonged use of HGH may lead to hypertrophy or enlargement of abdominal organs, further exacerbating the distension of the abdomen. However, there exists limited comprehensive empirical evidence concerning adverse outcomes associated with the misuse of GH in human subjects. The majority of reported instances detailing side effects are predominantly anecdotal in nature and are often intertwined with the misuse of multiple compounds. These potential consequences are posited to mirror those observed in cases of acromegaly, a condition characterized by excessive GH production, which may manifest as hypertension, carpal tunnel syndrome, diabetes, neuropathy, and a range of other maladies [10,15-18]. However, it is important to acknowledge that the potential for unidentified side effects exists in cases of excessive or inappropriate use, particularly when GH is combined with other agents or administered at elevated doses. In animal studies, the administration of supraphysiological doses of GH has been linked to organ enlargement, most notably cardiomegaly, a phenomenon analogous to that seen in acromegaly [19,20]. Furthermore, instances of edema, orthostatic hypotension, myositis, carpal tunnel syndrome, and gynecomastia have been documented in frail elderly individuals subjected to GH administration [21,22], while occurrences of carpal tunnel syndrome and hyperglycemia have been noted during GH administration in healthy adults [23].

IGF-1

Most attributes associated with the misuse of IGF-I are challenging to differentiate from those arising due to GH misuse, given that heightened GH levels also stimulate IGF-I production. Nonetheless, instances of hypoglycemia, seizures, jaw pain, myalgia, edema, headaches, augmented liver and kidney dimensions, and modified liver function, among other effects, have been documented subsequent to the administration of recombinant human IGF-I (rhIGF-I) [22,24,25,26]. Among the prevalent adverse reactions, erythema and lipohypertrophy at the injection site have been most frequently reported [24].

Insulin

Altered collagen synthesis with the use of insulin has been suggested as a potential mechanism underlying Palumboism. Fibroblasts are the primary cells responsible for the production of collagen. Collagen is a key component of connective tissues, including the fascia and muscles of the abdomen. Insulin increased collagen production by two- to threefold and total protein production by twofold in cultures of lung fibroblasts. In cardiac myocytes, insulin increases DNA and collagen synthesis in fibroblasts in a dose-dependent fashion, and any alterations in collagen synthesis or metabolism could result in structural changes, potentially leading to the characteristic abdominal protrusion seen in Palumboism. The proanabolic capabilities of insulin have been confirmed with the meta-analysis of 25 studies, which showed that it exerts its positive regulation of lean muscle mass principally via an anticatabolic effect in reducing muscle protein breakdown, rather than positively affecting the muscle protein synthesis [27].

Ultimately, pinpointing a specific agent to the development of Palumboism is rather difficult due to polypharmacy among users. The overwhelming majority of individuals who engage in the recreational use of anabolic agents commonly employ a combination of multiple substances. Substances frequently employed as adjuncts to anabolic-androgenic steroids (AAS) encompass a range of compounds including alcohol, amphetamine, caffeine, cannabinoids, clenbuterol, cocaine, codeine, creatine, ephedrine, erythropoietin, gamma hydroxybutyrate, GH, heroin, human chorionic gonadotropin, insulin, insulin-like growth factor-I (IGF-I), tamoxifen, tobacco, and numerous others [28,29,30]. In a United States-based study involving 41 individuals using insulin, it was revealed that 95% of these individuals concurrently utilized AAS and engaged in polypharmacy by incorporating an average of 16.2 ± 5.6 performance-enhancing drugs into their annual regimen [28].

## Conclusions

The available evidence on the pathophysiology of Palumboism is very limited. Basic science pathophysiology literature however points to a direct role of insulin and GH in the development of the condition. Furthermore, an anecdotal correlation exists between the prolonged use of high-dose anabolic steroids, particularly GH and IGF-1, and the development of Palumboism. This literature review is limited by the small number of available studies on the target pathology, which restricts the comprehensiveness and generalizability of the findings and underscores the urgent need for further research to build a more robust evidence base. Subsequent research is warranted to deepen our understanding of the pathophysiology of Palumboism and to explore additional contributing factors to this condition. The systematization of the available data has to be conducted to broaden the knowledge of complications, potential prevention, and treatment of Palumboism.
